# High incidence and reversible bradycardia events following alectinib initiation

**DOI:** 10.1111/1759-7714.14769

**Published:** 2022-12-19

**Authors:** Dongqi Yuan, Fuyi Zhu, Ran Zuo, Yu Wang, Gengwei Huo, Jinfang Cui, Ping Yue, Peng Chen

**Affiliations:** ^1^ Tianjin Medical University Cancer Institute and Hospital, National Clinical Research Center for Cancer, Tianjin’s Clinical Research Center for Cancer, Key Laboratory of Cancer Prevention and Therapy Tianjin China

**Keywords:** anaplastic lymphoma kinase, alectinib, bradycardia, non‐small‐cell lung cancer

## Abstract

**Background:**

With the widespread use of alectinib in patients with anaplastic lymphoma kinase (ALK)‐positive non‐small‐cell lung cancer (NSCLC), its cardiotoxicity has gradually emerged, including new‐onset sinus bradycardia (SB). However, the incidence, timing, severity, and risk factors of alectinib‐induced bradycardia remain unknown.

**Methods:**

From January 2020 to June 2022, 93 patients with ALK‐positive NSCLC treated with alectinib were enrolled in this retrospective analysis. These patients had heart rate (HR) recorded before and after alectinib administration. By reviewing electronic medical records and follow‐up, the HR changes of patients during medication were recorded. The potential risk factors associated with alectinib‐induced SB were explored.

**Results:**

According to an HR cut‐off of 60 beats per minute (bpm), 47 patients (50.54%) experienced at least one recorded bradycardia. The mean HR of total participants before alectinib administration was 78.32 (standard deviation [SD], 9.48) and after was 64.88 (SD, 12.21). The median maximum change in HR (range) for all patients was 11 (−55, +4) bpm. For the bradycardia subgroup, the HR of most patients (76.60%) hovered around 50–60 bpm, and 61.70% of SB occurred within 3 months after alectinib administration. Multivariate analysis indicated that baseline HR (odds ratio [OR] 0.86, 95% confidence interval [CI] 0.79–0.93, *p* < 0.001) and history of hypertension (OR 13.71, 95% CI 2.49–76.38, *p* = 0.003) were independent risk factors for alectinib‐related bradycardia.

**Conclusions:**

Alectinib‐induced bradycardia had a high incidence, appeared relatively early, and was reversible by dose reduction or withdrawal.

## Graphical abstract

To clarify the bradycardia associated with alectinib, 93 patients with anaplastic lymphoma kinase‐positive non‐small‐cell lung cancer treated with alectinib were enrolled in this retrospective analysis. The results showed that alectinib‐induced bradycardia had a high incidence, appeared relatively early, and was reversible by dose reduction or withdrawal. Baseline heart rate and history of hypertension were independent risk factors for alectinib‐related bradycardia.

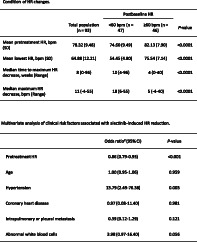

## INTRODUCTION

Alectinib, an oral, highly selective, second‐generation tyrosine kinase inhibitor (TKI), has dramatic efficacy in patients with anaplastic lymphoma kinase (ALK) rearrangement non‐small‐cell lung cancer (NSCLC).^1^ Three randomized clinical trials demonstrated that alectinib improved clinical effect and blood–brain barrier penetration compared with crizotinib in initial or treated patients with ALK‐rearranged.^2^, ^3^, ^4^ Alectinib is approved for first‐line treatment of patients with ALK‐positive advanced or metastatic NSCLC and second‐line treatment for disease progression after crizotinib.^5^ As a life‐saving therapy, despite alectinib's benefits, cardiovascular complication is gaining more attention.^6^, ^7^, ^8^ A study based on the pharmacovigilance database revealed the common alectinib‐associated cardiac disorders, including cardiac arrhythmias, heart failures, and pericardial and coronary artery disorders.^9^ During the development of ALK inhibitors, cardiovascular toxicities were not broadly recognized, but worries about heart rate (HR) decrease have gradually risen. The adverse effect of crizotinib causing sinus bradycardia (SB) in patients has been widely recognized.^10^, ^11^, ^12^, ^13^ ALK inhibitors are considered to have a class of effects leading to bradycardia. A meta‐analysis of 2915 participants from 12 clinical studies showed no significant difference in the incidence of bradycardia between alectinib and crizotinib.^6^ However, the initial clinical trials demonstrated different SB incidences in the alectinib treatment, such as 10.50% in ALEX, 0.01% in J‐ALEX, and 39.20% in ALESIA.^2^, ^3^, ^4^ The possible reasons for this difference are inconsistent definition of the threshold for bradycardia and bradycardia as an asymptomatic adverse reaction incompletely reported. Compared with clinical trials, patients in the real world tend to have worse performance status (PS) and more comorbidities, so adverse events might be more frequent and more serious. In addition, alectinib largely improves progression‐free survival (PFS) and overall survival in patients.^4^ Therefore we should keep an eye on the adverse events caused by the long‐term use of this agent. Cardiovascular complications are the main reason for death in individuals with cancer besides tumors, which seriously affect the quality of life and the time of survival, and there is increasing consideration of the cardiotoxicity related to targeted drugs.^14^, ^15^

To elucidate the incidence, severity, and risk factors of alectinib‐induced SB, we conducted a retrospective cohort study. We reviewed the electrocardiograms before and after medication, recorded the timing and degree of bradycardia, and documented the intrinsic clinical characteristics hoping to identify risk factors that may predispose patients to bradycardia.

## METHODS

### Study population

This study was a single‐center, retrospective cohort study performed at the Tianjin Medical University Cancer Institute and Hospital (TJMUCH), Tianjin, China. For patients with ALK rearrangement NSCLC treated with alectinib at TJMUCH from January 2020 through June 2022, we conducted electronic medical records queries and phone follow‐ups after local ethics committee approval. Inclusion criteria were adults ≥18 years of age with ALK‐positive NSCLC receiving alectinib monotherapy. HR and recognized risk factors for cardiovascular disease were recorded across time. This retrospective analysis included only patients with available pre‐treatment HR and post‐treatment HR measurements. We looked through the electronic medical records to extract risk factors, including baseline clinicopathological characteristics, comorbidities, hematologic parameters, potential symptoms related to SB, and treatment response. Patients with incomplete medical records were excluded.

### Outcomes

The primary outcome was new‐onset bradycardia after alectinib initiation; patients with preexistent bradycardia at baseline were excluded. Bradycardia was defined as a resting HR <60 beats per minute (bpm). The patient's HR were obtained from an electrocardiogram (ECG), ECG monitoring, electronic equipment, etc. We collected the time of the first onset of SB, the lowest HR recorded postbaseline, and the degree of HR decline. Bradycardia was documented and graded based on the National Cancer Institute Common Terminology Criteria for Adverse Events version 5.0 (CTCAE). The secondary outcomes were participants discontinuing medication owing to disease progression, intolerance of side reactions, or death for any reason. Response Evaluation Criteria in Solid Tumors version 1.1 was used to evaluate tumor response.

### Statistical analysis

Continuous variables use the mean ± standard deviation (SD) or median (interquartile range [IQR]) and categorical variables use the frequency counts with percentages for descriptive statistics. Difference analyses of baseline characteristics and cardiac parameters between bradycardia and nonbradycardia groups were conducted using Student's *t*‐test, Pearson's *χ* ^2^ test, or the Mann–Whitney U test in an appropriate way. Independent variables with *p* < 0.1 in the analysis of difference that may increase the risk of alectinib‐induced bradycardia were estimated using logistic regression analysis. The PFS of the total population and subgroups was assessed using the Kaplan–Meier method. All analyses were conducted in SPSS software (version 25.0) and two‐tailed *p* < 0.05 was considered statistically significant.

## RESULTS

### Patient characteristics

**FIGURE 1:**
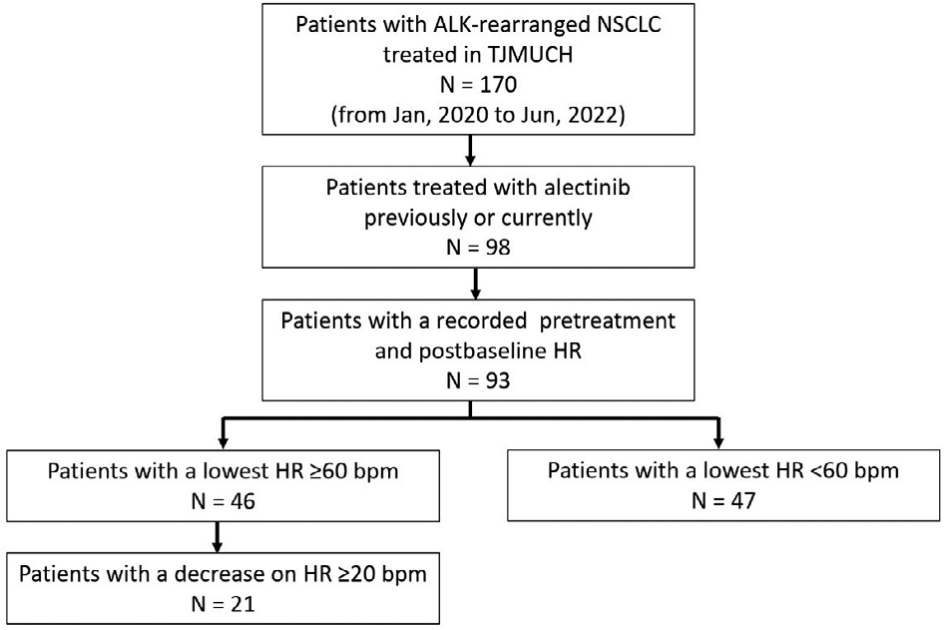
Study cohort. ALK, anaplastic lymphoma kinase; bpm, beats per minute; HR, heart rate; NSCLC, non‐small‐cell lung cancer; TJMUCH, Tianjin medical university Cancer Institute and Hospital.

From January 2020 to June 2022, 170 patients with ALK‐positive NSCLC presented to TJMUCH. A total of 98 patients were previously or currently treated with alectinib monotherapy, and five of them were excluded due to incomplete medical records. The screening process is shown in Figure 1. The participants had a mean age of 54.61 ± 10.70 years and a mean body mass index (BMI) of 24.75 ± 2.53 kg/m^2^. Fifty‐eight patients (62.37%) were female and 62 patients (66.67%) had a PS of 0 to 1 (Table 1). A total of 96.77% of patients were adenocarcinoma, 97.85% of patients had stage III or IV disease at alectinib initiation, and tumor metastases were common in bone (41.94%), lung or pleura (29.03%), and brain (25.81%). In addition, 39% (36 patients) received alectinib as first‐line systemic treatment and the median time of medication was 13 months (IQR 8.00–21.00 months) (Table 2). By the time of data analysis, 77.42% of patients (*n* = 72) were still using alectinib. One patient received first‐line treatment with alectinib in stage I because of her advanced age and intolerance to surgery. Another patient started alectinib in stage I for preoperative adjuvant therapy because the tumor was too close to the hilum for surgery.

**TABLE 1:** Baseline characteristics of patients

Variable	Total population (*n* = 93)	Postbaseline HR		*p* value
		<60 bpm (*n* = 47)	≥60 bpm (*n* = 46)	
Age at onset of therapy, mean (SD)	54.61 (10.70)	57.17 (11.50)	52.00 (9.22)	**0.019**
<60	55 (59.14)	19 (40.43)	36 (78.26)	**<0.001**
≥60	38 (40.86)	28 (59.57)	10 (21.74)	
Sex, *n* (%)				
Male	35 (37.63)	17 (36.17)	18 (39.13)	0.768
Female	58 (62.37)	30 (63.83)	28 (60.87)	
BMI (kg/m^2^), mean (SD)	24.75 (2.53)	25.05 (2.25)	24.44 (2.78)	0.243
18.5–24	38 (40.86)	16 (34.04)	22 (47.83)	0.176
≥24	55 (59.14)	31 (65.96)	24 (52.17)	
ECOG PS, *n* (%)				
0–1	62 (66.67)	29 (61.70)	33 (71.74)	0.305
≥2	31 (33.33)	18 (38.30)	13 (28.26)	
Smoking status, *n* (%)				
Former, current	24 (25.81)	9 (19.15)	15 (32.61)	0.138
Baseline comorbidities, *n* (%)				
Hypertension	20 (21.51)	15 (31.91)	5 (10.87)	**0.014**
Diabetes mellitus	7 (7.53)	4 (8.51)	3 (6.52)	0.716
Coronary heart disease	7 (7.53)	6 (12.77)	1 (2.17)	0.053
Thyroid disease	11 (11.83)	5 (10.64)	6 (13.04)	0.720
Baseline cardiovascular medications, *n* (%)	11 (11.83)	7 (14.89)	4 (8.70)	0.355

*Note*: Bold *p* values indicate statistically significant results.

Abbreviations: bpm, beats per minute; BMI, body mass index; ECOG PS, Eastern Cooperative Oncology Group performance status; HR, heart rate; SD, standard deviation.

**TABLE 2:** Baseline characteristics of disease

Variable	Postbaseline HR			*p* value
	Total population (*n* = 93)	<60 bpm (*n* = 47)	≥60 bpm (*n* = 46)	
Pathological type, *n* (%)				
Adenocarcinoma	90 (96.77)	44 (93.62)	46 (100.00)	0.082
Non‐adenocarcinoma	3 (3.23)	3 (6.38)	0 (0.00)	
Clinical stage, *n* (%)				
I	2 (2.15)	0 (0)	2 (4.35)	1.000
III	22 (23.66)	11 (23.40)	11 (23.91)	0.999
IV	69 (74.19)	36 (76.60)	33 (71.74)	0.999
Baseline disease metastasis, *n* (%)				
Brain	24 (25.81)	15 (31.91)	9 (19.57)	0.174
Bone	39 (41.94)	21 (44.68)	18 (39.13)	0.588
Intrapulmonary and pleural	27 (29.03)	10 (21.28)	17 (36.96)	0.096
Alectinib treatment line, *n* (%)				
First	36 (38.71)	17 (36.17)	19 (41.30)	0.611
Non‐first	57 (61.29)	30 (63.83)	27 (58.70)	
Second	28 (30.11)	16 (34.04)	12 (26.09)	
Third	25 (26.88)	13 (27.66)	12 (26.09)	
Fourth	3 (3.23)	1 (2.13)	2 (4.35)	
Fifth	1 (1.08)	0 (0.00)	1 (2.17)	
Median medication time (IQR)	13 (8.00, 21.00)	13 (8.00, 21.00)	13 (7.75, 20.00)	0.219

Abbreviations: bpm, beats per minute; HR, heart rate; IQR, interquartile range.

### Longitudinal changes in HR during alectinib treatment

**FIGURE 2:**
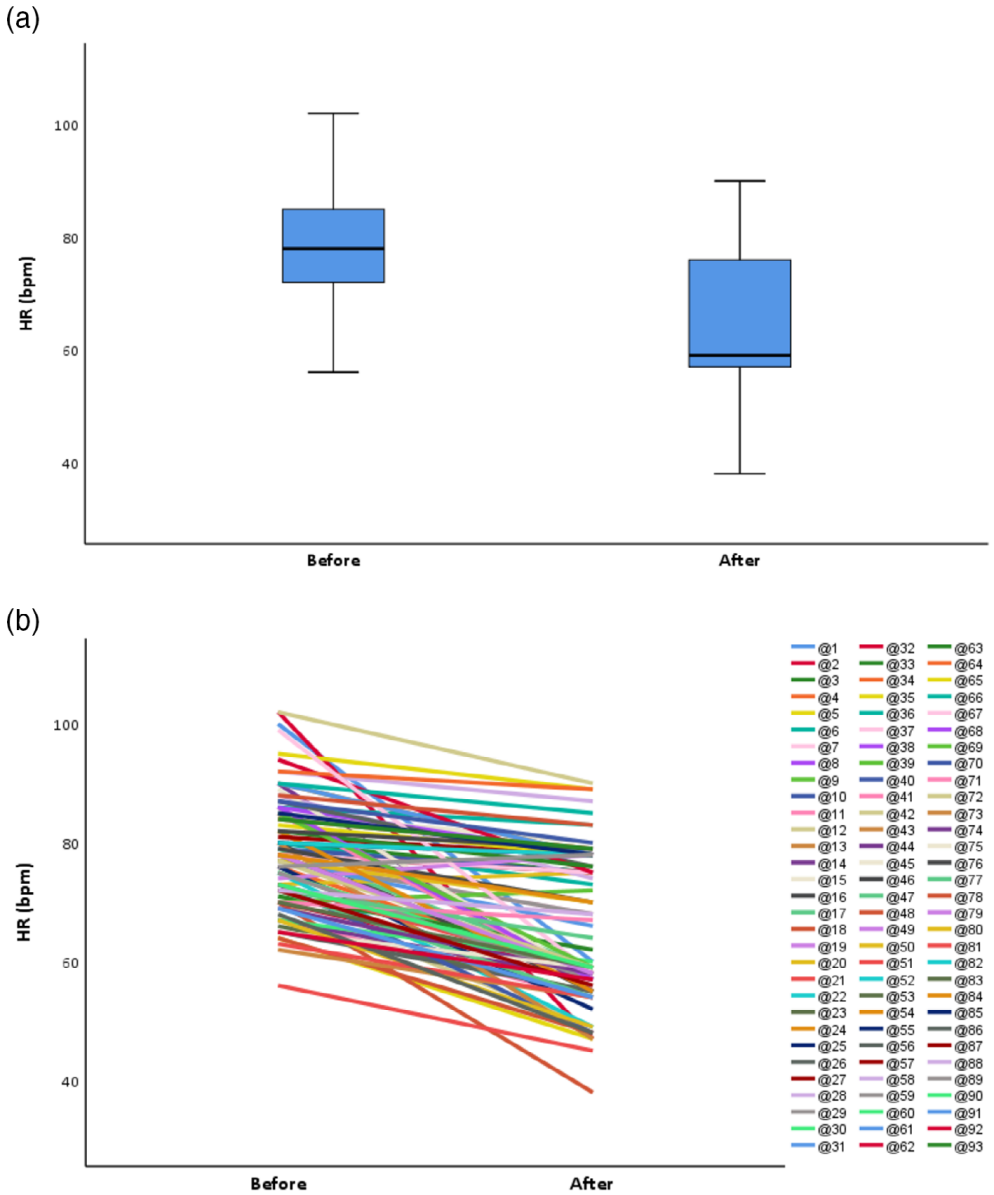
Changes in HR before and after alectinib in the overall population. HR, heart rate.

Given the HR is dynamic, we chose the lowest recorded HR as the grouping basis. According to an HR cut‐off of 60 bpm, 47 patients (50.54%) experienced at least one recorded bradycardia. The mean HR of total participants before the alectinib was 78.32 (SD, 9.48) and after the alectinib was 64.88 (SD 12.21) (Table 3). Figure 2 illustrates the magnitude of HR changes before and after alectinib. In particular, there was a significant difference in the pretreatment HR (*p* < 0.0001), the mean lowest HR achieved (*p* < 0.0001), and the median time to maximum HR decrease (*p* < 0.0001) between patients who did and did not experience SB (Table 3). The median maximum change in HR (range) for the overall population was 11 (−55, +4) bpm (Table 3). The median maximum decrease in HR was 18 bpm for the 47 patients with SB, which was significantly greater than for patients without SB (*p* < 0.0001) (Table 3). Furthermore, we performed a more in‐depth analysis of the patients in the bradycardia group. The SB group included more than half of patients (*n* = 29) who developed bradycardia within 3 months of alectinib initiation (Table 3). For the bradycardia subgroup, the HR of most patients (76.60%) hovered around 50–60 bpm (Table 3).

**TABLE 3:** Condition of heart rate (HR) changes

Variable	Postbaseline HR			*p* value
	Total population (*n* = 93)	<60 bpm (*n* = 47)	≥60 bpm (*n* = 46)	
Magnitude of HR changes				
Mean pretreatment HR, bpm (SD)	78.32 (9.48)	74.60 (9.49)	82.13 (7.90)	**<0.0001**
Mean lowest HR, bpm (SD)	64.88 (12.21)	54.45 (4.80)	75.54 (7.14)	**<0.0001**
Median time to maximum HR decrease, weeks (Range)	8 (0–96)	10 (4–96)	4 (0–40)	**<0.0001**
Median maximum HR decrease, bpm (Range)	11 (−4–55)	18 (6–55)	5 (−4–40)	**<0.0001**
Timing of SB, months, *n* (%)				
0–3	29 (31.18)	29 (61.70)		
3–6	14 (15.05)	14 (29.79)		
>6	4 (4.30)	4 (8.51)		
Degree of SB, bpm, *n* (%)				
50–60	36 (38.71)	36 (76.60)		
40–50	10 (10.75)	10 (21.28)		
30–40	1 (1.08)	1 (2.13)		

*Note*: Bold *p* values indicate statistically significant results.

Abbreviations: bpm, beats per minute; HR, heart rate; SB, sinus bradycardia; SD, standard deviation.

In the present study, we found two patients who developed bradycardia in combination with other types of arrhythmias. One patient, who had no arrhythmias before taking alectinib, developed SB after 10 months of dosing, atrial flutter after 12 months, and atrial premature beat after 14 months. One patient had complete right bundle branch block prior to drug administration and developed new‐onset atrial fibrillation combined with bradycardia 2 months after drug administration. Both patients were diagnosed with alectinib‐related cardiotoxicity excluding physiological and systemic diseases, cardiovascular disease, and interference from other drugs.

According to CTCAE, most bradycardia was in grade 1–2 and tolerable. Among the 47 patients with new‐onset bradycardia in this study, a total of three patients experienced discontinuation of treatment because of symptomatic bradycardia. One patient had HR fluctuations of 40–50 bpm during the drug administration, accompanied by chest tightness and weakness, and after 1 week of discontinuation the HR fluctuated to 60–70 bpm and the symptoms of chest tightness improved significantly. One patient's HR fluctuated between 40 and 50 bpm during the dosing period, accompanied by a significant increase in cardiac enzymes. The patient's HR then returned to normal and cardiac enzymes decreased after switching to lorlatinib. One patient stopped alectinib because of the lowest HR at 30–40 bpm and the HR increased to 50–60 bpm after discontinuation.

### Risk factors of alectinib‐induced HR reduction

We explored the potential risk factors of alectinib‐induced bradycardia. From Tables 1 and 2, we know that sex, BMI, Eastern Cooperative Oncology Group (ECOG) PS, smoking status, combined use of cardiovascular medications, baseline disease metastasis, and alectinib treatment line did not affect the probability of developing SB. Performing univariate analysis of hematological parameters, we can observe that abnormal liver function, abnormal hemoglobin, abnormal platelets, and dyslipidemia were not statistically different in the bradycardia and nonbradycardia groups (Table 4a). Furthermore, through the difference analysis of baseline cardiac parameters of 45 participants, we discovered the likelihood of developing bradycardia was not relevant to cardiac enzymes, brain natriuretic peptide, troponin I, and coagulation (Table 4b). Besides bradycardia, there are other common adverse effects while patients are taking alectinib, including constipation, edema, chest tightness, fatigue, and weight gain (Table 5). Difference analysis found that chest tightness and fatigue were more likely to occur in the bradycardia group (Table 5). According to univariate analysis, age (*p* = 0.019), history of hypertension (*p* = 0.014), abnormal baseline levels of white blood cell counts (*p* = 0.017), and decreased baseline HR (*p* < 0.001) were significantly related to alectinib‐induced bradycardia. Six clinical factors with *p* < 0.1 in univariate analysis were included in the logistic regression analysis. In a multivariate model (Table 6), however, only baseline HR (odds ratio [OR] 0.86, 95% confidence interval [CI] 0.79–0.93, *p* < 0.001) and history of hypertension (OR 13.71, 95% CI 2.49–76.38, *p* = 0.003) remained related to bradycardia development. Abnormal baseline white blood cell counts enhanced the potential to develop SB during alectinib treatment, while in multivariate logistic regression analysis this difference was not statistically significant.

**TABLE 4:** Baseline hematologic parameters of patients

(a)				
Variable	Postbaseline HR			*p* value
	Total population (*n* = 93)	<60 bpm (*n* = 47)	≥60 bpm (*n* = 46)	
Abnormal liver function, *n* (%)	46 (49.46)	25 (53.19)	21 (45.65)	0.467
Abnormal hemoglobin, *n* (%)	36 (38.71)	17 (36.17)	19 (41.30)	0.104
Abnormal white blood cells, *n* (%)	22 (23.66)	16 (31.04)	6 (13.04)	**0.017**
Abnormal platelet, *n* (%)	8 (8.60)	4 (8.51)	4 (8.70)	0.975
Dyslipidemia, *n* (%)	17 (18.28)	8 (17.02)	9 (19.57)	0.751

(b)				
Variable	Postbaseline HR			*p* value
	Total population (*n* = 45)	<60 bpm (*n* = 20)	≥60 bpm (*n* = 25)	
Abnormal cardiac enzymes, *n* (%)	14 (31.11)	6 (30.00)	8 (32.00)	0.885
Abnormal BNP or cTnI, *n* (%)	3 (6.67)	1 (5.00)	2 (8.00)	0.688
Abnormal coagulation, *n* (%)	24 (53.55)	12 (60.00)	12 (48.00)	0.423

*Note*: Bold *p* values indicate statistically significant results.

Abbreviations: bpm, beats per minute; BNP, brain natriuretic peptide; cTnI, cardiac troponin I; HR, heart rate.

**TABLE 5:** Adverse effects and general conditions after taking alectinib

Variable	Postbaseline HR			*p* value
	Total population (*n* = 93)	<60 bpm (*n* = 47)	≥60 bpm (*n* = 46)	
Adverse events, *n* (%)				
Constipate	65 (69.89)	35 (74.47)	30 (65.22)	0.331
Edema	41 (44.09)	19 (40.43)	22 (47.83)	0.472
Chest tightness	19 (20.43)	15 (31.91)	4 (8.70)	**0.005**
Fatigue	44(47.31)	29(61.70)	15(32.61)	**0.005**
Sleep, *n* (%)				
Excellent	27 (29.03)	5 (10.64)	22 (47.83)	1.000
Good	56 (60.22)	36 (76.60)	20 (43.48)	**<0.001**
Average	7 (7.53)	4 (8.51)	3 (6.52)	0.052
Poor	3 (3.23)	2 (4.26)	1 (2.17)	0.100
Appetite, *n* (%)				
Excellent	32 (34.41)	10 (21.28)	22 (47.83)	1.000
Good	48 (51.61)	26 (55.32)	22 (47.83)	**0.046**
Average	12 (12.90)	10 (21.28)	2 (4.35)	**0.005**
Poor	1 (1.08)	1 (2.13)	0 (0.00)	1.000
Weight, *n* (%)				
Usual	36 (38.71)	17 (36.17)	19 (41.30)	1.000
Increase	52 (55.91)	27 (57.45)	25 (54.35)	0.665
Decrease	5 (5.38)	3 (6.38)	2 (4.35)	0.595

*Note*: Bold *p* values indicate statistically significant results.

Abbreviations: bpm, beats per minute; HR, heart rate.

**TABLE 6:** Multivariate analysis of clinical risk factors associated with alectinib‐induced HR reduction

Variable	OR (95% CI)	*p* value
Pretreatment HR	0.86 (0.79–0.93)	**<0.001**
Age	1.00 (0.95–1.06)	0.959
Hypertension	13.79 (2.49–76.38)	**0.003**
Coronary heart disease	0.97 (0.08–11.40)	0.981
Intrapulmonary or pleural metastasis	0.39 (0.12–1.29)	0.121
Abnormal white blood cells	3.98 (0.97–16.40)	0.056

*Note*: Bold *p* values indicate statistically significant results.

Abbreviations: CI, confidence interval; HR, heart rate; OR, odds ratio.

### Treatment outcomes of alectinib

**FIGURE 3:**
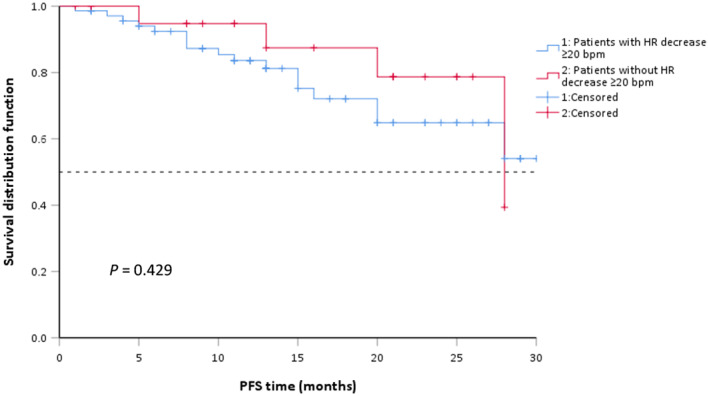
PFS by presence/absence of HR decrease ≥20 bpm. bpm, beats per minute; HR, heart rate; PFS, progression‐free survival.

To evaluate alectinib's clinical efficacy in our cohort study, we followed up with the enrolled patients and collected information on survival data. As shown in Table 7, 29 patients (31.18%) had partial response and 46.24% of patients had stable disease on alectinib. Until the last follow‐up, 21 patients progressed on alectinib or died of any reason (Table 7). There was no significant association between bradycardia and clinical efficacy (*p* = 0.687) (Table 7). Overall median PFS was not reached. We scrutinized whether there was a statistical difference in PFS between patients with an HR drop of ≥20 bpm and those with an HR drop of <20 bpm after alectinib initiation. A total of 22 participants (23.66%) experienced an HR reduction ≥20 bpm and 71 participants (76.34%) did not experience such a decrease. The subgroup of patients with a ≥20 bpm decline in HR had a median PFS of 28 months (95% CI 15.69–39.31), while those without a ≥20 bpm decline in HR did not reach median PFS (Figure 3). The difference was not statistically significant (*p* = 0.429).

**TABLE 7:** Treatment efficacy of alectinib

Variable	Postbaseline HR			*p* value
	Total population (*n* = 93)	<60 bpm (*n* = 47)	≥60 bpm (*n* = 46)	
Treatment efficacy, *n* (%)				0.687
PR	29 (31.18)	16 (34.04)	13 (28.16)	
SD	43 (46.24)	22 (46.81)	21 (45.65)	
PD/death	21 (22.58)	9 (19.15)	12 (26.09)	

*Abbreviations*: bpm, beats per minute; HR, heart rate; PR, partial response; PD, progressive disease; SD, stable disease.

## DISCUSSION

Through the analysis of longitudinal changes in HR after alectinib initiation, our study revealed that alectinib‐induced bradycardia had a high incidence, occurring in nearly half of patients, appeared relatively early, generally within 3 months of medication, and was reversible by dose reduction or withdrawal. Continuation of alectinib did not result in a progressive decrease in HR, indicating bradycardia was not cumulative toxicity. Because of its remarkable clinical efficacy, alectinib has an increasing number of users with a long duration of drug use. Our observation comprehensively described alectinib‐induced bradycardia, which is of particular significance given the absence of early markers to evaluate cardiotoxicity.

Patients with lower HR showed a higher likelihood of developing alectinib‐related bradycardia in our cohort. Ou et al. reported similar conclusions in a study of risk factors for crizotinib‐related bradycardia, indicating that HR <70 bpm was an independent risk factor of SB.^11^ Previous literature suggests that age, ECOG PS, use of beta‐blockers, etc. may be risk factors for crizotinib‐related bradycardia,^11^ but no association between these factors and alectinib‐related bradycardia was found in this study. Because our retrospective analysis only included participants with healthy HR at baseline, the cardiac adverse effects of alectinib in patients with preexisting severe bradycardia, such as HR <50 bpm, remain unclear. Considering a history of hypertension and baseline HR are strong predictors of alectinib‐related bradycardia, the use of alectinib in patients with comorbid hypertension and HR <50 bpm should pay caution. With the aging of patients with lung cancer and more cardiac complications in clinical practice,^16^ cardiac history and baseline cardiac function must be considered when administering alectinib.

The SB grading criteria specified by the CTCAE are not based on measured HR values but rely on symptoms and the need for hospitalization. A total of 47 participants reported alectinib‐related SB: 65.96% grade 1, 29.79% grade 2, and 4.26% grade 3. Chest tightness, fatigue, and edema are common symptoms in patients with cardiac dysfunction. In our cohort, 19 patients (20.43%) showed chest tightness, 44 patients (47.31%) showed fatigue, and 41 patients (44.09%) showed edema. Univariate analysis showed that chest tightness and fatigue are statistically associated with bradycardia. However, the causal relationship between chest tightness, fatigue, and bradycardia remains unclear. Nowadays, physicians are not adequately aware of the cardiotoxicity of alectinib and often neglect regular ECG monitoring during drug administration. This finding also suggests that when patients complain of chest tightness and weakness, in addition to considering the disease itself, we should also pay attention to the occurrence of alectinib‐induced cardiotoxicity. In our research, alectinib‐induced bradycardia appeared to be reversible, and three patients had a rapid return to normal heart rate after discontinuation of the drug for severe bradycardia. Similarly, a recent case report also showed that a patient with NSCLC developed bradycardia after the application of alectinib, and the patient returned to sinus rhythm after withdrawal of the drug.^17^ The main cardiovascular adverse event of the third‐generation ALK inhibitor lorlatinib was hyperlipidemia. The incidence of hypercholesterolemia was 82.4% and of hypertriglyceridemia was 60.7%, and 81% of patients required lipid‐lowering medication during lorlatinib administration.^18^ The close link between blood lipid levels and cardiovascular disease has been widely recognized.^19^ No significant difference in baseline lipid levels between the bradycardia and nonbradycardia groups was found in our retrospective cohort. Considering that in the present observation 55.91% of patients experienced weight gain during treatment, clinicians need to keep an eye on changes in blood lipid levels.

Alectinib‐induced bradycardia provides further evidence to prove the association between alectinib initiation and adverse cardiovascular outcomes. Cardiovascular toxicity associated with ALK inhibitors has caught the attention of investigators. To evaluate the relationship between ECG parameters and corresponding drug concentrations, Morcos conducted a retrospective analysis of two clinical studies of alectinib, NP28761 and NP28673, pointing out that alectinib‐related cardiovascular toxicity was dose‐irrelevant, and the average HR dropped by 11–13 bpm in the 2 weeks after medication.^7^ A recent pharmacovigilance analysis brought targeted therapy‐related cardiac toxicities in patients with NSCLC to light, indicating that ALK inhibitors with higher odds cause QT prolongation and conduction disease.^20^ Compared with the available published randomized trial data, our present examination showed higher odds of alectinib‐induced HR reduction with a 50.54% incidence of SB.^2^, ^3^, ^4^ Although unsure, the reasons for this discrepancy might be the systematic medical records, comprehensive follow‐ups, patients with more comorbidities and worse PS, while using the HR cut‐off value of 60 bpm to define bradycardia. In addition to crizotinib and alectinib, other small‐molecule targeted drugs, such as the vascular endothelial growth factor (VEGF) inhibitor pazopanib, the break point cluster region‐Abelson (BCR‐ABL) inhibitor nilotinib, and the MAP/ERK kinase (MEK) inhibitor trametinib, have also reported bradycardia as treatment‐related common cardiac adverse reactions.^21^ Although drugs are evaluated for cardiotoxicity before marketing, the specificity of oncology drugs, such as the lack of data from healthy subjects and the difficulity of achieving the required overexposure, results in the detection of their cardiotoxicity often being delayed.^22^

At present, there is no consensus on the pathogenesis of ALK inhibitor‐related arrhythmia, and plausible mechanisms that involve ion signaling pathways, endocrine regulation, and drug metabolism may underlie this condition. Ou et al. pointed out that crizotinib‐induced arrhythmia may be associated with the antagonism of L‐calcium channels, the effect of epithelial‐mesenchymal transition, and the function of sinoatrial and atrioventricular nodes.^13^ In vitro experiments in mice, Doherty et al. found that the cardiotoxicity of crizotinib may be associated with reactive oxygen species (ROS) increase, caspase activation, cholesterol accumulation, and inhibition of potassium, sodium, and calcium channels.^23^ The ion channels mechanism continues to be a priority of study in ALK inhibitor‐related HR reduction, but an association with alectinib has not been identified. Thyroid hormone receptors exist in cardiomyocytes and vascular endothelial cells, which are extremely sensitive to changes in thyroid hormone levels in the blood. Even in the stage of subclinical hypothyroidism, there are adverse effects on the cardiovascular system, such as reducing HR and cardiac contractility and increasing vascular resistance.^24^ A prospective study evaluating thyroid dysfunction caused by TKIs in NSCLC patients showed that the incidence of thyroid dysfunction was about 8%, and most occurred 1 month after TKI treatment.^25^ Therefore, clinicians should be aware that hypothyroidism may lead to bradycardia and regular monitoring of thyroid function in patients taking ALK inhibitors is necessary. The patients enrolled in this study were not routinely tested for thyroid function, and the relationship between alectinib and patient thyroid function needs to be verified by trials. In addition, ALK inhibitors can cause hypogonadism and decreased testosterone levels may be associated with the development of bradycardia.^26^ ALK inhibitors are primary metabolized by CYP3A4/5 enzymes in the liver cytochrome P450 (CYP) family. Strong inhibitors of CYP3A4/5 may increase the plasma concentration of ALK inhibitors and aggravate their cardiotoxicity. Bradycardia was found to be a pharmacodynamic response to crizotinib, with an average decline in HR of 2.5 bpm for every 100 ng/ml increase in plasma concentration.^27^ However, alectinib and its main active metabolite 4 (M4) are less dependent on CYP3A4, suggesting a lower risk of drug–drug interactions with alectinib.^28^ Cancer patients may be accompanied by cardiac diseases, taking other cardiotoxic drugs, as well as electrolyte disturbances and vagus nerve excitation caused by drug side effects, all of which can cause patients to exhibit impaired cardiac function. In the present observation, there was no statistically significant connection between cardiovascular medications and the development of new bradycardia (*p* = 0.355). Due to the available data on beta­blockers and non‐dihydropyridine calcium channel blockers (CCBs) being limited, physicians should be cautious about combining alectinib with such agents. There is no consensus on the exact mechanism of ALK‐TKI‐induced arrhythmia in patients with advanced NSCLC. To elucidate the molecular mechanisms of TKI‐related arrhythmia, additional mechanistic and prospective studies are required to reveal undiscovered common targets and common biological pathways.

Several limitations should be recognized. Our research is a single‐center retrospective cohort study with limited participants and nonuniformly available data. Due to the lack of a pre‐specified, standardized cardiovascular monitoring and follow‐up protocol, we are missing changes in ECG parameters other than HR, and the potential to recognise additional events was limited. Selection bias may exist in patients who received at least one ECG after alectinib administration. Patients who accepted more cardiovascular monitoring had higher odds of detecting medication‐related arrhythmia. Electrocardiogram follow‐up times varied, potentially leading to extra detection bias. Furthermore, given the insufficient awareness of the severity of bradycardia, some SB and the management after SB are possibly undocumented in medical records. Finally, some research variables have not been clearly defined, such as the classification of sleep and diet.

In summary, alectinib is linked to a significantly raised risk of newly developed SB, which is reversible and dose‐independent. Although alectinib‐related bradycardia is mostly asymptomatic and not life‐threatening, there are patients who encounter dose reduction, drug discontinuation, or even death due to severe bradycardia. The newly prospective combination of alectinib and the anti‐angiogenic agent bevacizumab has obtained positive results, along with the risk of cardiovascular adverse events increasing.^29^ When this therapy mode is practiced in patients, clinicians need to pay attention to the cardiovascular toxicity. Considering the increasing number of people taking alectinib, profound exploration to characterize the mechanisms and treatment of alectinib‐related SB is significant. With the collaboration of oncologists and cardiologists, along with increased awareness and knowledge, an optimal administration process should be formulated to guarantee the maximum survival benefit of the patient.

## AUTHOR CONTRIBUTIONS

Peng Chen and Dong‐qi Yuan were responsible for the conception and design of the study. Dong‐qi Yuan, Fu‐yi Zhu, Ran Zuo, Yu Wang, and Geng‐wei Huo were responsible for collecting data. Dong‐qi Yuan, Jin‐fang Cui, and Ping Yue analyzed the data. All authors were responsible for the revision and approval of the final version of the manuscript.

## CONFLICT OF INTEREST

The authors declared no conflicts of interest for this work or regarding the publication of this paper.

## FUNDING INFORMATION

This work was supported by funding from the Tianjin Major Disease Prevention and Control Science and Technology Project, Tianjin Municipal Science and Technology Bureau (18ZXDBSY00050).
